# Delineation of Homeostatic Immune Signatures Defining Viremic Non-progression in HIV-1 Infection

**DOI:** 10.3389/fimmu.2020.00182

**Published:** 2020-03-05

**Authors:** Amit Kumar Singh, Sukeshani Salwe, Varsha Padwal, Shilpa Velhal, Jyoti Sutar, Shilpa Bhowmick, Srabani Mukherjee, Vidya Nagar, Priya Patil, Vainav Patel

**Affiliations:** ^1^Department of Biochemistry and Virology, Indian Council of Medical Research (ICMR)-National Institute for Research in Reproductive Health, Mumbai, India; ^2^Department of Molecular Endocrinology, Indian Council of Medical Research (ICMR)-National Institute for Research in Reproductive Health, Mumbai, India; ^3^Department of Medicine, Grant Medical College & Sir J. J. Group of Hospitals, Mumbai, India

**Keywords:** viremic non-progressors, long-term non-progressors, HIV-1, homeostasis, viral pathogenesis, disease progression, immune activation, CD4+ central memory

## Abstract

Viremic non-progressors (VNPs), a distinct group of HIV-1-infected individuals, exhibit no signs of disease progression and maintain persistently elevated CD4+ T cell counts for several years despite high viral replication. Comprehensive characterization of homeostatic cellular immune signatures in VNPs can provide unique insights into mechanisms responsible for coping with viral pathogenesis as well as identifying strategies for immune restoration under clinically relevant settings such as antiretroviral therapy (ART) failure. We report a novel homeostatic signature in VNPs, the preservation of the central memory CD4+ T cell (CD4+ T_*CM*_) compartment. In addition, CD4+ T_CM_ preservation was supported by ongoing interleukin-7 (IL-7)-mediated thymic repopulation of naive CD4+ T cells leading to intact CD4+ T cell homeostasis in VNPs. Regulatory T cell (Treg) expansion was found to be a function of preserved CD4+ T cell count and CD4+ T cell activation independent of disease status. However, in light of continual depletion of CD4+ T cell count in progressors but not in VNPs, Tregs appear to be involved in lack of disease progression despite high viremia. In addition to these homeostatic mechanisms resisting CD4+ T cell depletion in VNPs, a relative diminution of terminally differentiated effector subset was observed exclusively in these individuals that might ameliorate consequences of high viral replication. VNPs also shared signatures of impaired CD8+ T cell cytotoxic function with progressors evidenced by increased exhaustion (PD-1 upregulation) and CD127 (IL-7Rα) downregulation contributing to persistent viremia. Thus, the homeostatic immune signatures reported in our study suggest a complex multifactorial mechanism accounting for non-progression in VNPs.

## Introduction

Human immunodeficiency virus (HIV) infection causes progressive depletion of CD4+ T cells through rampant viral replication (1, 2), ensuing impairment of cellular immunity and ultimately susceptibility to opportunistic infections (3, 4). In the majority of people living with HIV (PLHIV), this necessitates antiretroviral therapy (ART) to suppress viremia and halt progression to AIDS (5). However, long-term non-progressors (LTNPs), a minority of HIV-1-infected individuals, do not show signs of progression (6). LTNPs have been shown to maintain preserved CD4+ T cell counts and symptom-free survival in the absence of ART for several years (≥7 years). The introduction of highly sensitive plasma viral load (VL) measurement showed that some LTNPs have low or undetectable plasma viremia and can be classified into elite controllers (ECs) and viremic controllers (VCs) based on their ability to suppress viral replication with plasma VL <50 copies/ml and 50–2,000 copies/ml, respectively (7). Interestingly, another cohort of LTNPs has been identified with preserved CD4+ T cell counts despite a high level of viral replication (>2,000 HIV-1 RNA copies/ml) for several years, termed viremic non-progressors (VNPs) (8, 9).

HIV-1 infection in VNPs resembles simian immunodeficiency virus (SIV) infection of natural hosts like sooty mangabeys (SMs) and African green monkeys (AGMs) in that chronically infected SM and AGM maintain stable CD4+ T cell counts despite a high level of viral replication. An additional feature of SIV infection of natural hosts is low peripheral immune activation (10). However, there are conflicting reports with respect to levels of immune activation in VNPs (11–14). Also, preservation of CD4+ T cell counts over extended periods of infection in the face of ongoing viral replication presents an opportunity to evaluate T cell homeostasis and perturbations in T cell dynamics within these individuals compared to those with typical progression. Very limited data have suggested that VNPs may share features of disease progression with progressors as well as harbor specific pathogenic signatures such as preservation of CD4+ stem cell central (T_SCM_) memory and preferential resistance to infection of CD4+ T_SCM_ and CD4+ T_CM_ subsets (14)_._ The influence of preserved CD4+ T cell counts and thus putatively retained “CD4 help” on CD8+ memory/effector T cell homeostasis in these individuals also remains unexplored (15–17).

Delineation of unique characteristics of VNPs in halting immune deficiency despite high viral replication may provide valuable insights into immune compensatory mechanisms employed to cope with viral pathogenesis, a scenario that may develop under ART through acquired or transmitted resistance. Thus, in this study, our goal was to obtain a comprehensive homeostatic characterization of both CD4+ and CD8+ T cell compartments in VNPs to define unique signatures associated with non-progression. These immune signatures may provide novel strategies for disease management and immune restoration following therapy.

## Materials and Methods

### Study Groups

**Figure 1:**
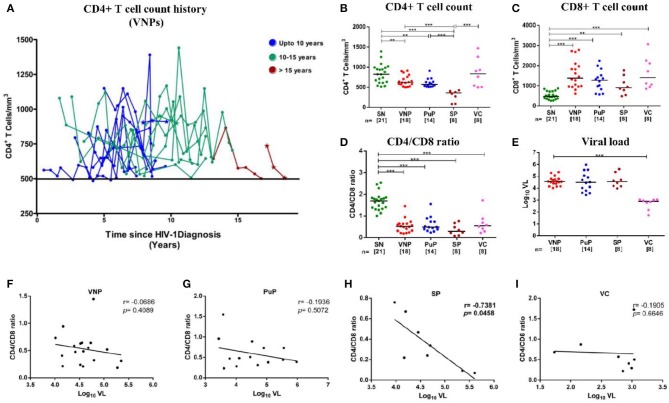
Distribution of absolute CD4+ T cell count history in viremic non-progressors (VNPs) and clinical, immunological, and virological characteristics of the study groups. **(A)** 18 VNPs were recruited following stringent criteria. The cutoff absolute count of 500 cells/mm^3^ is represented by the solid line. The last time point, for each sample, is the time of recruitment of participants for the study. Each line represents one individual. Individuals with <6 data points are represented as a *(*n* = 3). Color represents years of infection until recruitment for the study: Blue, 7–10 years; Green, 10–15 years; red, >15 years. **(B,C)** Absolute T cell count in blood as measured by flow cytometry. **(B)** Absolute CD4+ T cell count. **(C)** Absolute CD8+ T cell count. **(D)** CD4/CD8 ratio calculated from absolute CD4+ and CD8+ T cell count. **(E)** Plasma viral load (Log_10_ VL). **(F–I)** Correlation between CD4/CD8 ratio and plasma viral load in **(F)** VNPs, **(G)** putative progressors (PuPs), **(H)** standard progressors (SPs), and **(I)** viremic controllers (VCs). Comparisons between groups were calculated by Mann–Whitney non-parametric test (*p < 0.05; **p < 0.01; ***p < 0.001). *p* and *r* values for associations were determined by Spearman's correlation test, with linear regression shown as a line. Significant (*p* < 0.05) values are in bold.

Twenty-one HIV seronegative (SN) individuals and 48 HIV-1-infected participants were recruited for this study from ART Center at Grant Medical College & Sir J. J. Group of Hospitals, Mumbai. Peripheral blood was collected, and a signed informed consent was obtained from the recruited participants in accordance with ICMR-NIRRH Institutional Ethics Committee for Clinical Studies (Project no. 225/2012) recommendations. The protocols were approved by ICMR-NIRRH Institutional Ethics Committee Review Board. Recruited participants were screened by HIV TRI-DOT test to confirm HIV-1 seropositivity. HIV-1-infected participants were classified as VNPs (*n* = 18) and putative progressors (PuPs, *n* = 14) based on duration of infection, absolute CD4+ T cell counts, and VL. We recruited VNPs, considering risk of AIDS events in individuals with CD4+ T cell counts <500 cells/mm^3^ (18, 19), with a sustained history of CD4+ T cell counts ≥500 cells/mm^3^ (Figure 1A). We also recruited VNPs with VL of >10,000 copies/ml to avoid the possible impact of host restriction factors like HLA-B^*^27/HLA-B^*^57 on viral replication (20). Thus, as summarized in Table 1, VNPs were recruited as per the above described criteria and ≥7 years of infection since HIV-1 diagnosis with no history of any ensuing coinfections. Recently infected (6 months−3 years from the date of HIV-1 diagnosis) therapy-naive participants with CD4+ T cell counts ≥500 cells/mm^3^ and apparently non-controlled viral replication (VL > 2,000 copies/ml) were also recruited, termed putative progressors (PuPs). These were matched to VNPs in terms of CD4+ T cell counts to avoid the effects of immunological impairment that follow extensive CD4+ T cell depletion, thus enabling comparison of T cell dynamics with similar levels of CD4+ T cell counts (immune competence). During the course of recruitment, viremic controllers (VCs, *n* = 8) and standard progressors (SPs, *n* = 8) were also identified and incorporated. VCs differed from VNPs only with respect to VL being ≤2,000 copies/ml, while SPs differed from PuPs only with respect to CD4+ T cell counts being <500 cells/mm^3^. The clinical characteristics of all the recruited participants are summarized in Table 1. All the recruited participants were ART naive.

**Table 1:** Clinical, immunological, and viral characteristics of the study population.

	**Seronegative Individuals** **[SN, *n* = 21]**	**Viremic Non-progressors** **[VNPs, *n* = 18]**	**Putative progressors** **[PuPs, *n* = 14]**	**Standard progressors** **[SPs, *n* = 8]**	**Viremic controllers** **[VCs, *n* = 8]**
Age^a^ (years), Range	44 (24–59)	40 (18–54)	36 (21–59)	44 (32–55)	43 (29–60)
Gender	Female = 10 Male = 11	Female = 14 Male = 4	Female = 7 Male = 7	Female = 0 Male = 8	Female = 5 Male = 3
CD4 count^a^ (cells/mm^3^), Range	878 (513–1,289)	624 (501–910)	570 (510–908)	354 (82–424)	900 (501–1,253)
Viral load^a^,^b^ (Log_10_ copies/ml), Range	NA	4.60 (4.01–5.35)	4.49 (3.44–5.98)	4.55 (3.97–5.60)	2.90 (1.73–3.04)
Duration of infection^a^ (years), Range	NA	10 (7–18)	1 (0.5–03)	0.8 (0.5–03)	10 (8–24)
Antiretroviral therapy status	NA	Naive	Naive	Naive	Naive
HLA-B*27/B*57 status^c^	Not tested	HLA-B*27 (+ve = 1/14) and HLA-B*57 (+ve = 1/17)	HLA-B*27 (+ve = 3/12), HLA-B*57 (+ve = 1/13)^d^	HLA-B*27 (+ve = 1/8) and HLA-B*57 (+ve = 0/8)	HLA-B*27 (+ve = 1/7) and HLA-B*57 (+ve = 1/8)

a *Data are expressed as the median (range)*.

b *Viral load was estimated at the time of sampling*.

c HLA-B*27 and HLA-B*57 allele status of seven and two of the HIV-1-infected participants, respectively, was not available due to lack of SSP-PCR amplification.

d Only one participant was positive for both HLA-B*27 and HLA-B*57 allele. NA, not applicable.

### Assays for Absolute CD4+, CD8+ T Cell Count, and Plasma Viral Load

Fresh ethylenediaminetetraacetic acid (EDTA)-stabilized blood was used for enumeration of absolute CD4+ and CD8+ T cell counts. Absolute T cell count was determined using four-color flow cytometry with BD Multitest antibody cocktail and liquid counting beads following stain/lyse/no-wash protocol. Data acquisition was performed on BD ACCURI C6 flow cytometer (BD Biosciences), and data analysis was carried out on FlowJo (Tree Star Inc., Oregon, USA).

Viral nucleic acid was isolated from plasma samples using MagNA Pure Compact Nucleic Acid Isolation kit (Roche Diagnostics, New Jersey, USA) with MagNA Pure Compact instrument. VL (copies per milliliter) was quantified using COBAS TaqMan 48 Analyzer (Roche) with detection limit of 34 copies/ml of plasma.

### Immunophenotyping

For phenotypic characterization, immunostaining of 200 μl of fresh peripheral whole blood with four-color combinations of the following fluorescently labeled monoclonal antibodies, anti-CD3 (Clone:SK7), anti-CD4 (Clone: RPA-T4), anti-CD8 (Clone: SK1), anti-CD25 (Clone: M-A251), anti-CD127 (Clone: HIL-7R-M21), anti-CCR7 (Clone: 150503), anti-CD45RA (Clone: HI100), anti-HLA-DR (Clone: L243), anti-CD38-PECY5 (Clone: HIT2), anti-PD-1 (Clone: EH12.1), anti-CD28 (Clone: 28.2), and anti-CD31 (Clone: WM59), was performed as reported previously (21). Briefly, 200 μl of fresh EDTA blood was incubated with fluorescently labeled monoclonal antibodies for 20 min at room temperature. Erythrocytes were lysed using FACS lysing solution (BD Biosciences) and washed twice with staining buffer [phosphate-buffered saline (PBS) with 0.2% fetal bovine serum (FBS)]. Data acquisition was performed on BD ACCURI C6 flow cytometer (BD Biosciences) where at least 50,000 events gated on lymphocyte population were acquired. Data analysis was carried out using FlowJo (Tree Star Inc., Oregon, USA). Anti-mouse Ig, κ/Negative Control compensation beads (BD Biosciences) were used to set compensation parameters. Fluorescence minus one (FMO) control was used to identify and gate cells.

### Plasma Interleukin-7 Measurement

Peripheral blood was centrifuged, and plasma was stored at −80°C until used. Interleukin-7 (IL-7) was measured in thawed plasma samples using Quantikine HS human IL-7 ELISA kit (R & D Systems, Milan, Italy) according to the manufacturer's recommendations.

### Sample Genotyping

Genomic DNA was extracted from peripheral blood mononuclear cells (PBMCs) using the QIAamp DNA minikit (Qiagen, Valencia, CA), according to manufacturer's instructions. The quality of DNA was assessed by spectrophotometry. HLA genotyping was performed by PCR amplification using low-resolution sequence-specific primer (SSP) method for both HLA-B^*^27 and HLA-B^*^57 using HLA-ready gene kits (Inno-Train Diagnostik GmbH, Kronberg, Taunus, Germany), according to manufacturer's instructions.

Genotyping of viral *vpr* and *env* HIV-1 genes was performed by Sanger sequencing of proviral DNA amplified by nested PCR (detailed in Supplementary Method 1). Bidirectional Sanger sequencing was performed on ABI 3730XL sequencer. Electropherograms obtained post sequencing were examined and edited with ABI sequence scanner V1 (Applied Biosystems). Sequence contigs were generated with CAP3 implementation in BioEdit v7.25 (22). Quality assessment and hypermutation analysis were performed with QC tools available on LANL HIV database (https://www.hiv.lanl.gov/content/sequence/QC/index.html). Codon-wise multiple sequence alignments were generated with Gene-Cutter (https://www.hiv.lanl.gov/content/sequence/GENE_CUTTER/cutter-help.html). Co-receptor tropism was predicted using PhenoSeq (http://tools.burnet.edu.au/phenoseq/), WebPSSM (23–25), and Geno2Pheno (coreceptor) 2.5 (https://coreceptor.geno2pheno.org/). Further, N-linked glycosylation sites present in the *env* sequences were predicted with the webtool “N-GlycoSite” (26).

### Statistical Analysis

Statistical analysis was performed on GraphPad Prism software (San Diego, California, USA). The data are represented as scatter plots, and bars indicate median values. Mann–Whitney non-parametric test was performed for comparison between different study groups. Bivariate associations were determined by Spearman's rank correlation test. For all statistical calculations, *p* < 0.05 was considered significant.

## Results

### Clinical, Immunological, and Virological Characteristics of the Study Population

A total of 69 participants were recruited for this cross-sectional study, including 21 HIV SN individuals and 48 therapy-naive HIV-1-infected individuals (Table 1). Infected individuals were classified as VNPs (*n* = 18), PuPs (*n* = 14), SPs (*n* = 8), and VCs (*n* = 8). As described in Table 1, we found no significant age difference among study groups. We also observed, as expected, female gender being overrepresented in non-progressors, that is, both VNPs and VCs (8), while participants in the SP group were exclusively male. VNPs and VCs, by definition, were infected for significantly longer duration [10 years (7–18 years); 10 years (8–24 years), respectively] compared to PuPs and SPs [1 year (0.5–3 years); 10 months (0.5–3 years), respectively]. HLA-B^*^27 and HLA-B^*^57 are key host genetic factors known to be associated with slower disease progression and control of viral replication. Six HIV-1-infected participants were positive for HLA-B^*^27 allele (6/41, 14.6%) and three HIV-1-infected participants were positive for HLA-B^*^57 allele (3/46, 6.5%). However, we observed no enriched HLA-B^*^27/HLA-B^*^57 in any of our study groups.

All infected groups except VCs had significantly lower CD4+ T cell counts compared to SN individuals (Figure 1B). Interestingly, VCs, along with other infected groups, showed a significant expansion of CD8+T cells and inversion of CD4/CD8 ratio (<1.0), a more robust indicator of disease progression (27), compared to SN group in spite of elevated (similar to SN) CD4+T cell counts and low viremia (Figures 1C–E). Importantly, when VL was correlated with either CD4+ T cell counts (Supplementary Figures S2A–D) or CD4/CD8 ratio, no correlation was observed in either VNPs or PuPs, suggesting a lack of overt immune impairment but a significant negative correlation was observed in SPs as expected (Figures 1F–I). Thus, VNPs and PuPs had similar profiles of these parameters, viz. CD4+ T cell counts, CD8+ T cell counts, CD4/CD8 ratio, and VL, enabling a robust comparison between these two groups for homeostatic parameters.

### Immune Activation and CD4+ Regulatory T Cells

**Figure 2:**
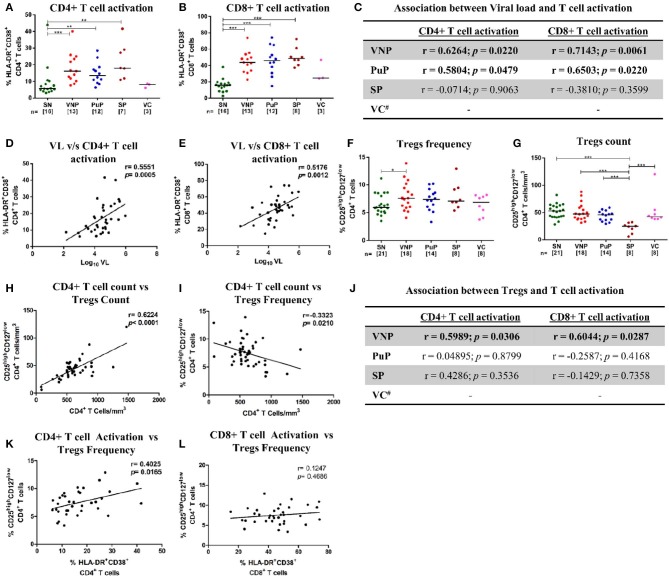
Immune activation and CD4+ regulatory T cells (Tregs). **(A,B)** Frequency of activated T cells for all the study groups. **(A)** CD4+ T cell activation. **(B)** CD8+ T cell activation. **(C)** Correlation of CD4+ and CD8+ T cell activation with plasma viral load in viremic non-progressors (VNPs), putative progressors (PuPs), and standard progressors (SPs). **(D,E)** Cumulative correlation analysis across all HIV-1-infected individuals of CD4+T cell activation **(D)** and CD8+ T cell activation **(E)** with plasma viral load. **(F)** Treg frequency. **(G)** Treg count based on absolute CD4+ T cell count. **(H,I)** Cumulative correlation analysis across all HIV-1-infected individuals of Treg count **(H)** and Treg frequency **(I)** with absolute CD4+ T cell count. **(J)** Association of CD4+ and CD8+ T cell activation with Treg frequency, respectively, in VNPs, PuPs, and SPs. **(K,L)** Cumulative correlation analysis across all HIV-1-infected individuals of CD4+T cell activation **(K)** and CD8+ T cell activation **(L)** with Treg frequency. Comparisons between groups were calculated by Mann–Whitney nonparametric test (*p < 0.05; **p < 0.01; ***p < 0.001). *p* and r values for associations were determined by Spearman's correlation test, with linear regression shown as a line. Significant (*p* < 0.05) values are in bold. ^#^T cell activation data were available for only three VCs and were not further analyzed **(C,J)**.

Chronic immune activation is a key driving force for CD4+ T cell depletion and progression to AIDS. To ascertain the role of immune activation in the protective phenotype of VNPs, CD4+ and CD8+ T cell activation was evaluated by measuring co-expression of CD38 and HLA-DR (CD3+CD8-CD38+HLA-DR+ and CD3+CD8+CD38+HLA-DR+, respectively) on T cells (28) (Supplementary Figure S1A). All infected individuals except VCs had elevated levels of immune activation across both CD4+ and CD8+ T cell compartments compared to SN individuals (Figures 2A,B). Although T cell activation data of only three VCs were available, VCs had similar levels of CD4+ and CD8+ T cell activation compared to SN individuals, reflective of their relatively low viremia (Figures 2A,B). The elevated level of T cell activation in both CD4+ and CD8+ T cell compartments positively correlated with plasma VL in both VNPs and PuPs (Figure 2C). SPs did not show such association, most likely due to a small sample size (Figure 2C). However, cumulative analysis of all infected individuals revealed that both CD4+ and CD8+ T cell activation correlated with viremia independent of disease status (Figures 2D,E). Subsequently, we evaluated CD4+ regulatory T cells (Tregs) in these groups due to their known suppressive effect on T cell activation and effector cell function and generation (29, 30). Expression of CD25 (IL-2Rα) and CD127 (IL-7Rα) on T cells was used to enumerate Tregs (CD3+CD4+CD25^high^CD127^low^) (31, 32) (Supplementary Figure S1B). All infected groups, including VCs, showed a higher frequency of Tregs compared to SN individuals (*p* = 0.0486 for VNPs) (Figure 2F). However, on comparison of Treg counts, we noticed that VNPs, PuPs, and VCs, groups with high CD4+ T cell counts, also had a preserved pool of Tregs, while SPs, the only group with severe CD4+ T cell depletion, exhibited Treg count depletion as well (Figure 2G). Congruently, absolute CD4+ T cell count correlated positively with Treg count (*p* < 0.0001) and negatively with Treg frequency (*p* = 0.0210) (Figures 2H,I). Next, while the level of both CD4+ and CD8+ T cell activation positively correlated with Treg frequency in VNPs only (Figure 2J), cumulative (of all infected individuals) analysis revealed a positive correlation of Treg frequency with CD4+ T cell activation (*p* = 0.0165) but not with CD8+ T cell activation (Figures 2K,L). Of note, VCs, known to have lower immune activation and elevated CD4+ T cell counts, also had high Treg counts. In summation, observed Treg expansion and preserved Treg counts in VNPs are dependent on preserved CD4+ T cell counts as well as activation of this subset. Thus, maintained Treg pools, observed in VNPs, would not be sustained in PuPs following continued CD4+ T cell depletion.

### Preservation of Homeostatic Distribution of CD4+ T Cell Subsets in Viremic Non-progressors

We investigated the distribution of memory/naive subsets, reflective of homeostasis within the CD4+ and CD8+ T cell compartments across HIV-1-infected groups compared to SN individuals. The evaluation of expression of CD45RA and/or CCR7 on T cells enabled identification of four functionally distinct populations in CD4+ (CD3+CD4+) and CD8+ (CD3+CD4-) T cell compartments: naive (T_N_, CD45RA+CCR7+), central memory (T_CM_, CD45RA-CCR7+), effector memory (T_EM_, CD45RA-CCR7-), and terminally differentiated (T_TD_, CD45RA+CCR7-) (33) (Supplementary Figure S1B).

**Figure 3:**
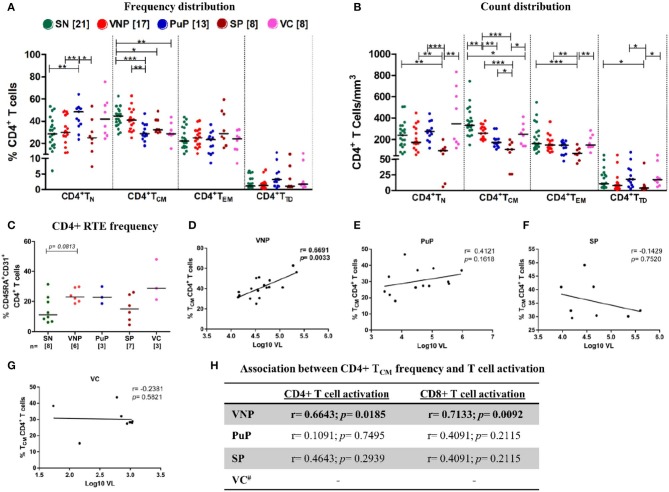
Viremic non-progressors (VNPs) maintain CD4+ T cell homeostasis by increased thymic production of naive CD4+ T cells and preservation of CD4+ T_CM_ subset. **(A,B)** Distribution of CD4+T cell subsets into naive (T_N_), central memory (T_CM_), effector memory (T_EM_), and terminally differentiated (T_TD_). **(A)** Frequency of CD4+ T cell subsets. **(B)** Absolute count of CD4+ T cell subsets based on absolute CD4+ T cell counts. **(C)** CD4+ recent thymic emigrant (RTE) frequency. **(D–G)** Correlation between CD4+ T_CM_ frequency and plasma viral load in **(D)** VNPs, **(E)** putative progressors (PuPs), **(F)** standard progressors (SPs), and **(G)** viremic controllers (VCs). **(H)** Association between CD4+ T_CM_ frequency and T cell activation (CD4+ and CD8+) in VNPs, PuPs, SPs, and VCs. Comparisons between groups were calculated by Mann–Whitney non-parametric test (*p < 0.05; **p < 0.01; ***p < 0.001). *p* and r values for associations were determined by Spearman's correlation test, with linear regression shown as a line. Significant *p-*values (*p* < 0.05) are in bold. ^#^T cell activation data were available for only three VCs and were not further analyzed **(H)**.

When we evaluated the naive CD4+ T cell compartment (CD4+ T_N_), VNPs and SN individuals had similar CD4+ T_N_ frequencies, while PuPs had significantly increased CD4+ T_N_ frequencies compared to both VNPs and SN individuals (*p* = 0.0065 and *p* = 0.0027, respectively) (Figure 3A). However, both PuPs and VNPs had similar levels of CD4+ T_N_ count compared to SN individuals (Figure 3B), suggesting the contribution of alterations in other subsets to the increased frequency of these cells in PuPs. As expected, SPs with decreased CD4+ T_N_ frequency also had significant depletion of CD4+ T_N_ counts (Figures 3A,B), reflective of systemic CD4+T cell depletion. On the other hand, VCs, a protected group, had similar CD4+ T_N_ frequencies and counts compared to SN individuals (Figures 3A,B). It was noteworthy to observe maintained CD4+ T_N_ compartment in VNPs despite an extended duration of infection with a high viral replication. Thus, we explored the role of thymic output in terms of recent thymic emigrants (RTEs), a population of naive CD4+ T cells recently derived from thymus, in our study groups (34) (Supplementary Figure S1B). We observed a trend toward increased CD4+ RTEs in VNPs compared to SN individuals (*p* = 0.0813) despite an extended duration of infection (Figure 3C), suggesting a role for increased thymic output in the maintenance of the naive CD4+ T cell compartment and CD4+ T cell pool within these individuals.

The CD4+ central memory compartment (CD4+ T_CM_) represents a critical hub for both generalized immune function as well as HIV pathogenesis as these are the cells most often infected by the virus (35). Intriguingly, VNPs exhibited similar CD4+ T_CM_ frequencies and limited, though significant, depletion of CD4+ T_CM_ counts compared to SN individuals (*p* = 0.0058) (Figures 3A,B). Furthermore, VNPs had significantly higher CD4+ T_CM_ frequencies and counts compared to PuPs despite an almost 10-fold higher duration of infection (Figures 3A,B). These findings indicated the presence of mechanisms ensuring preservation of the CD4+ T_CM_ compartment in VNPs. We next sought to investigate the association of CD4+ T_CM_ subset with T cell activation and VL to assess the lack of disease progression despite ongoing viral replication in VNPs. Surprisingly, we observed a significant positive correlation between frequency (and count, Supplementary Figures S3A–D) of CD4+ T_CM_ and VL only in VNPs (*p* = 0.0033) (Figures 3D–G) and also observed a significant positive correlation between frequency of activated CD4+ and CD8+ T cells compared to frequency of CD4+ T_CM_ (*p* = 0.0185 and *p* = 0.0092, respectively) (Figure 3H). However, no such correlation was found for PuPs. CD4+ T_EM_ frequencies and CD4+ T_EM_ counts were similar across the groups except in the case of SPs that showed a significant depletion of CD4+ T_EM_ counts (Figures 3A,B). CD4+ T_TD_ frequencies and CD4+ T_TD_ counts were also similar across all the study groups (Figures 3A,B).

In summation, our data highlighted preserved CD4+ T_CM_ homeostasis in the face of extensive viremia-driven immune activation in VNPs.

### VNPs Demonstrate Altered Homeostatic Relationships in the CD8+ T Cell Compartment

**Figure 4:**
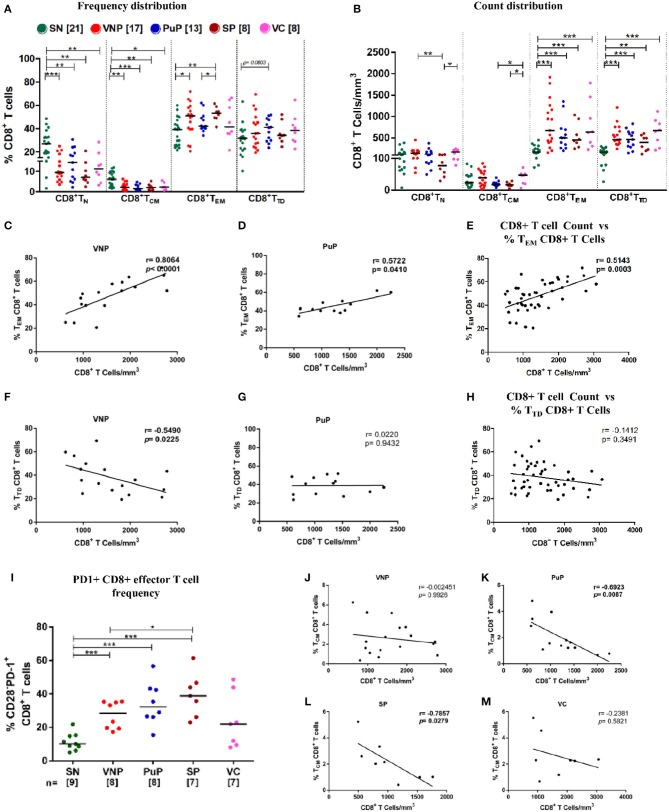
Viremic non-progressors (VNPs) preserve CD8+ T_CM_ cell number and expand total effector CD8+ T cell pool. **(A,B)** Distribution of CD8+ T cell subsets into naive (T_N_), central memory (T_CM_), effector memory (T_EM_), and terminally differentiated (T_TD_). **(A)** Frequency of CD8+ T cell subsets. **(B)** CD8+ T cell subsets count based on absolute CD8+ T cell counts. **(C,D)** Correlation of CD8+ T_EM_ cell frequency with absolute CD8+ T cell counts, respectively, in **(C)** VNPs and **(D)** putative progressors (PuPs). **(E)** Cumulative correlation analysis across all HIV-1-infected individuals of CD8+ T_EM_ cell frequency with absolute CD8+ T cell count. **(F,G)** Correlation of CD8+ T_TD_ cell frequency with absolute CD8+ T cell counts respectively in **(F)** VNPs and **(G)** PuPs. **(H)** Cumulative correlation analysis across all HIV-1-infected individuals of CD8+ T_TD_ cell frequency with absolute CD8+ T cell count. **(I)** Frequency of exhausted (PD1+) CD8+ effector T cells. **(J,M)** Correlation of CD8+ T_CM_ cell frequency with absolute CD8+ T cell counts in **(J)** VNPs, **(K)** PuPs, **(L)** standard progressors (SPs), and **(M)** viremic controllers (VCs). Comparisons between groups were calculated by Mann–Whitney nonparametric test (*p < 0.05; **p < 0.01; ***p < 0.001). *p* and r values for associations were determined by Spearman's correlation test, with linear regression shown as a line. Significant *p-*values (*p* < 0.05) are in bold.

Cytotoxic T lymphocytes (CTLs) are critical in eliminating HIV-infected cells and thus contributing to set point viremia in HIV infection (36–38). These cells are derived from long-lived precursors (memory CD8+ T cells) with capacity to undergo rapid and robust proliferation on antigenic exposure (39). We investigated distribution of memory/effector subsets in CD8+ T cell compartment to get a deeper insight into non-progression in VNPs (Supplementary Figure S1B). In spite of systemically increased CD8+ T cell count (Figure 1C), we observed significantly decreased CD8+ T_N_ frequencies, but preserved absolute counts, across all infected groups compared to SN individuals (Figures 4A,B). SPs were the only infected group that showed significant depletion of CD8+ T_N_ counts (Figure 4B).

As shown in Figure 4B, all infected groups had significantly higher counts of total CD8+ effector T cells (CD8+T_EM_ and CD8+ T_TD_) compared to SN individuals which was reflected in the observed increased absolute CD8+ T cell counts shown in Figure 1C. On evaluation of CD8+ effector T cell compartment individually, in terms of CD8+ T_EM_ and CD8+ T_TD_ frequency distribution, significantly higher frequencies of CD8+ T_EM_ in VNPs and SPs compared to SN individuals (*p* = 0.0266 and *p* = 0.0027, respectively) were observed but not in PuPs and VCs (Figure 4A). In this regard, CD8+ T_EM_ frequency positively correlated with absolute CD8+ T cell count in both VNPs and PuPs (*p* < 0.0001 and *p* = 0.0410) (Figures 4C,D). However, no such correlation was observed for SPs and VCs (possibly due to the small sample size) (Supplementary Figures S4A–D). Indeed, cumulative analysis of all infected individuals revealed a positive correlation between absolute CD8+ T cell count and CD8+ T_EM_ frequency (*p* = 0.0003; Figure 4E). Subsequently, a strong trend suggestive of increased CD8+ T_TD_ frequencies was observed only in PuPs compared to SN individuals (*p* = 0.0603) (Figure 4A). Surprisingly, CD8+ T_TD_ frequency negatively correlated with absolute CD8+ T cell count in VNPs (*p* = 0.0225) but not in any other infected group (Figures 4F,G). Also, cumulative analysis with all infected individuals did not show a correlation between absolute CD8+ T cell count and CD8+ T_TD_ frequency (Figure 4H). Taken together with the aforementioned negative correlation observed only in VNPs, it is likely that despite expansion of total CD8+ effector T cell counts, a relative diminution of CD8+ T_TD_ frequency was unique to VNPs. These observations suggest that a relatively restrained generation of terminally differentiated CD8+ T cells may play a role in viremic non-progression.

Considering the well-documented expansion of exhausted CD8+ effector T cells that accompany chronic HIV viral infection, we examined the frequency of exhausted effector cells (CD3+CD4-CD28-PD1+) within the circulating CD8+ T cell compartment (40) (Supplementary Figure S1B). We observed a significant increase in PD-1 expression on CD8+ effector T cells in VNPs, PuPs, and SPs compared to SN individuals (Figure 4I). No difference was observed in PD-1 expression on CD8+ effector T cells in VCs compared to SN individuals. While VNPs, probably due to significant chronic viremia, had increased levels of exhausted CD8+ effector T cells, it is intriguing to note that these levels were comparable and possibly lower than for PuPs and SPs in spite of a 10-fold longer duration of infection.

Furthermore, comparison of CD8+ T_CM_ subset revealed decreased CD8+ T_CM_ frequencies in all the infected individuals compared to SN individuals, but no significant depletion of CD8+ T_CM_ counts for any of the groups including SPs (Figures 4A,B). Subsequently, only progressors, PuPs and SPs, showed a negative correlation of CD8+ T_CM_ frequency with absolute CD8+ T cell count (Figures 4J–M). VNPs and VCs, on the other hand, showed a positive correlation of CD8+ T_CM_ count with absolute CD8+ T cell count (Supplementary Figures S4E–H).

### Deficient IL-7/IL-7R Levels With Limited Capacity to Facilitate Thymic Expansion

IL-7 plays crucial role in maintenance of T cell homeostasis, impacting development, proliferation, and survival. IL-7 responsiveness is dependent on the presence or absence of IL-7 receptor (IL-7R), comprising of IL-7-specific α chain (IL-7Rα)/CD127 and a common γ chain (γ_c_)/CD132, present on most T cells. Thus, IL-7R expression and circulating IL-7 levels were evaluated among study groups.

**Figure 5:**
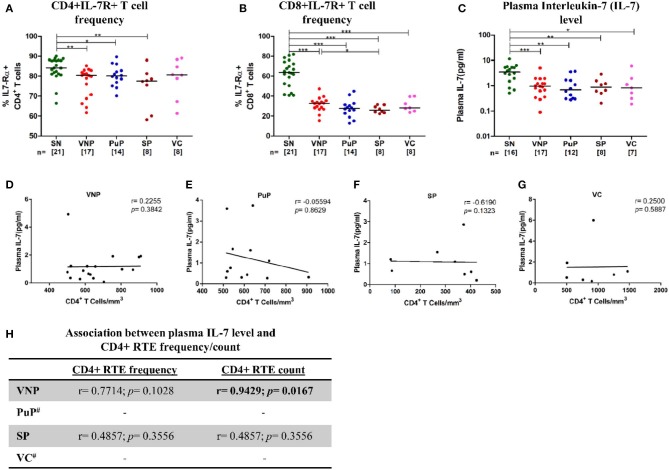
Deficient interleukin-7 (IL-7)/IL-7R levels with limited capacity to facilitate thymic expansion. **(A)** IL7-Rα expression on CD4+ T cells. **(B)** IL7-Rα expression on CD8+ T cells. **(C)** Plasma IL-7 levels in study groups. **(D–G)** Correlation of absolute CD4+ T cell count with plasma IL-7 level in **(D)** viremic non-progressors (VNPs), **(E)** putative progressors (PuPs), **(F)** standard progressors (SPs), and **(G)** viremic controllers (VCs). **(H)** Correlation of CD4+ recent thymic emigrants (RTEs) with plasma IL-7 level in VNPs, PuPs, SPs, and VCs. Comparisons between groups were calculated by Mann–Whitney nonparametric test (*p < 0.05; **p < 0.01; ***p < 0.001). *p* and r values for associations were determined by Spearman's correlation test, with linear regression shown as a line. Significant *p-*values (*p* < 0.05) are in bold. ^#^CD4+ RTE data were available for only three individuals for both PuPs and VCs and were not further analyzed **(H)**.

Surprisingly, despite relatively high CD4+ T cell counts, VNPs displayed significantly decreased frequency of IL7-Rα+ CD4+ T cells compared to SN individuals (Figure 5A). This decrease in frequencies of IL7-Rα+ CD4+ T cells was also observed in all other infected groups. The deficiency in IL-7R expression was even more apparent in all infected groups on CD8+ T cells compared to SN individuals (Figure 5B). Further, significantly reduced circulating IL-7 level was observed in all infected groups compared to SN individuals (Figure 5C). Interestingly, VNPs and all the other infected groups lacked the negative correlation between absolute CD4+ T cell count and plasma IL-7 level, a marker of severe CD4+ T cell depletion (<200 cells/mm^3^) (41, 42) (Figures 5D–G). However, we observed a positive correlation of plasma IL-7 level with CD4+ RTEs frequency/count (*p* = 0.0167) (Figure 5H) in VNPs, suggesting a limited capacity of IL-7-driven CD4+ RTE production and thus CD4+ T cell maintenance.

### Viral Genotypic Analysis

To identify non-attenuating viral signatures possibly associated with the VNP phenotype, we performed genotypic analysis of proviral DNA derived from PBMCs of a subset of recruited participants from all study groups. *vpr* gene was amplified from 8 VNPs, 9 PuPs, 6 SPs, and 4 VCs, while partial *env* gp120 (V1-V3 region) was amplified and assessed from 4 VNPs, 11 PuPs, 4 VCs, and 6 SPs (Supplementary Table S1). All the sequences were confirmed to be of HIV-1 subtype C and submitted to GenBank. Vpr genotyping revealed that Q3R, a mutation associated with reduced cytopathicity (43) of HIV-1 was only observed in two participants, one of the eight VNPs and one of the nine PuPs. Mutation R77Q, associated with impairment of T lymphocyte apoptosis and thereby delayed progression (44), was observed in five of the eight VNPs, four of the nine PuPs, as well as five of the six SPs. This mutation was not observed in any of the VCs assessed. Mutation F72L, associated with absence of nuclear import of pre-integration complex was observed in only one participant belonging to the VNP group. Highly conserved C terminal motif SRIG was also observed to be mutated in only two participants, one of the nine PuPs and one of the four VCs. Mutations W54G, R36W, I64E, L67A, I70S, L64PAR, and Q65R, all previously associated with disease progression, were not observed in any of the analyzed samples. Co-receptor tropism was also assessed by sequence analysis of gp120 (V1-V3) region with three algorithms viz., Geno2Pheno, WebPSSM, and Phenoseq. All the sequences isolated were predicted to be R5 tropic irrespective of the study group. Also, no significant group-specific changes were observed in the N-linked glycosylation patterns. Overall, while we did observe some *vpr* mutations previously shown to be associated with disease progression but unreported from India, their distribution was not found to be group specific or enriched in VNPs (summarized in Supplementary Table S2).

## Discussion

ART has enabled a reduction in HIV transmission, a prolonged life span, and an improved quality of life for PLHIV (45). However, limitations of lifelong adherence to therapy including drug resistance, medication-induced adverse events, and serious non-AIDS events have highlighted the need for a functional cure of infection that aims at achieving long-term drug-free remission in HIV-infected individuals in the absence of an efficacious vaccine or curative strategy (46, 47). VNPs are a rare group of HIV-1-infected individuals characterized by non-progression despite intensive viral replication. Defining mechanisms of resistance to immune deficiency in VNPs may lead to better understanding of key determinants of CD4+ T cell depletion and HIV pathogenesis that may instruct the aforementioned approach.

Previous cohort studies have established the advantage of high CD4+ T cell counts (≥500 cells/mm^3^) in minimizing the risk of comorbidities in HIV-infected individuals independent of viral replication (18, 19). In the current study, VNPs were compared to a stringently defined PuP group with similar CD4+ T cell count (≥500 cells/mm^3^) and viremia. The similar level of immune health, despite a drastic difference in the duration of infection in these groups, thus provided a window of opportunity to delineate established homeostatic immune signatures of viremic non-progression.

In this study, we found that HIV-1-infected VNPs had elevated levels of immune activation in both CD4+ and CD8+ T cell compartments comparable to progressors (PuPs and SPs). This finding is in stark contrast with a well-reported paradigm of low immune activation associated with disease non-progression in both HIV-1 infection of humans and non-pathogenic SIV infection of non-human primates (NHPs) (48, 49). Immune mechanisms with the ability to mitigate the effects of immune activation may thus be critical in maintaining viremic non-progression. Tregs are one of the key regulators of systemic immune activation, HIV-specific immune responses and are promising targets for immunotherapeutic modalities (50–52). Indeed, all groups except SPs had a preserved Treg compartment. A positive association of Treg count and a negative association of Treg frequency with absolute CD4+ T cell count were also found. Thus, Treg homeostasis was found to rely on the preservation of CD4+ T cell count, which was consistent with a previous finding, that in addition also showed that a low Treg count adversely affects Treg function (53). Moreover, as also previously reported, a positive association of Treg frequency with CD4+ T cell activation but not CD8+ T cell activation was observed (29, 54). Taken together, Treg dynamics and function seem to be a consequence of both preserved CD4+ T cell count and CD4+ T cell activation, features of viremic non-progression observed in our study. Considering that VNPs and not PuPs have the ability to maintain preserved CD4+ T cell counts, the latter would be expected to progress due to the breakdown of Treg-mediated compensatory mechanisms that are in operation in VNPs. To be noted however, Treg expansion seems insufficient to suppress systemic immune activation in VNPs, as previously reported in studies of untreated chronic HIV infection associated with high viral replication [reviewed in Chevalier and Weiss (55)]. In contrast to our study, Roider et al. (56) has delineated a possible role for Treg-mediated suppression of immune activation in a pediatric cohort resembling VNPs. The discordance in the relationship between Tregs and immune activation observed in VNPs and pediatric slow progressors may reflect the disparate immune responses to viral infection observed in a developing *vis-à-vis* mature immune system (57). Treg levels have also been shown to suppress autologous HIV-specific responses (58–60). Previous studies by Thorborn et al. (61, 62) have demonstrated increased sensitivity of CD4+CD25- effectors toward Treg-mediated suppression in asymptomatic HIV-1 infection. In summation, activation-induced Treg expansion in VNPs may perhaps result in the suppression of HIV-specific effector responses contributing to high viremia.

Previous studies in NHPs and humans have strongly implicated CD4+ T_CM_ preservation as an important determinant of maintenance/reconstitution of CD4+ T cell counts following SIV/HIV infection (63, 64). Interestingly, in our study, VNPs exhibited a direct positive association of CD4+ T_CM_ cell preservation with both the key markers of HIV pathogenesis, immune activation, and VL. Our data thus indicate the presence of intact mechanisms of CD4+ T_CM_ subset stability in VNPs despite years of viremic infection as opposed to progressive depletion of this subset in PuPs. These may include mechanisms, as described above, to avert activation-induced depletion of the CD4+ T_CM_ compartment, which in turn may preserve and enhance the restorative capacity following infection. This privileged status of CD4+ T_CM_ cells was also reported in earlier NHP studies (65), revealing the association of CD4+ T_CM_ preservation with long-term survival of vaccinated SIV-infected macaques who initially controlled viremia but experienced viral breakthrough eventually. This finding highlighted the importance of a robust control of acute infection in CD4+ T_CM_ preservation for long-term survival despite a high viral replication. A similar course of infection, although not verified, could have operated in our cohort of VNPs. Selective resistance of CD4+ T_CM_ cells to HIV-1 infection has also been implicated as a mechanism of CD4+ T_CM_ cell preservation in VNPs (14). However, these data are contradicted by earlier reports that do not show any discriminatory infection of this subset in a similar cohort of non-HLAB^*^27/B^*^57 LTNPs with high viremia (20). Also, the CD4+ T_CM_ subset has been well-established to be the preferential circulating reservoir in chronic HIV-1 infection (35, 66).

Antiviral CD8+ T cell immunity could be another principal contributor in protection from disease progression in VNPs. However, despite increased absolute CD8+ T cell counts, we observed no apparent control of viral replication in VNPs. Furthermore, VNPs have been reported to exhibit similar HIV-specific CD8+ T cell responses compared to chronic progressors (67). Even though potent antiviral CD8+ T cell-mediated immunity seems unlikely in VNPs, a role for the expanded CD8+ T cell compartment in the establishment of virus–host equilibrium is apparent (68). It is also important to note in our study that the generation of CD8+ T cells in VNPs seems under appropriate CD4 help in the form of maintained CD4+ T_CM_ cell homeostasis, which is required for priming and induction of functional memory CD8+ T cells (16, 17, 69). CD8+ T_CM_ cells have limited effector function but can readily proliferate and differentiate into effector cells on antigenic stimulation (70). Our study revealed that a stable CD8+ T_CM_ compartment is maintained independent of disease status. Further, as expected, elevated CD8+ T cell counts, across all the infected groups, were driven by expansion of both effector memory CD8 (T_EM_) and terminally differentiated CD8 (T_TD_) T cells. T cell exhaustion is marked by increased expression of inhibitory receptor PD-1, a negative regulator of immune activation (71). In VNPs, expansion of CD8+ effector T cells under persistent antigenic stimulation leads to increased PD-1 expression as was also observed for progressors. Thus, accumulation of exhausted CD8+ effector T cells in conjugation with Treg-mediated suppression, in VNPs, may be associated with impairment of effector functions in the control of viral replication. Interestingly, evaluation of frequency distribution of CD8+ T cell compartment and association analysis revealed a diminished CD8+ T_TD_ cell expansion [compared to effector memory CD8 (T_EM_)] in VNPs that suggests disparate regulation in the generation of differentiated effectors. The contribution of this signature, however, in sustaining the apparent virus–host equilibrium remains unclear.

The current study also revealed similar frequency and counts of naive CD4+ T cells in VNPs compared to SN individuals, while a significant increase in frequency (but not count) was observed in PuPs, suggesting a significant depletion of non-naive subsets in this group. The preservation of naive CD4+ T cell pool was consistent with increased thymic function observed in both VNPs and PuPs compared to SN individuals. Cytokine-driven expansion is also instrumental in T cell homeostasis (72). IL-7 plays a critical role in the survival and maintenance of naive T cell number (73). Surprisingly, plasma IL-7 level was found to be decreased across all the study groups independent of viremia. However, responsiveness of IL-7 depends on the expression of the IL-7 receptor (IL-7R) on the target cell surface. Here, we demonstrated that similar to progressors, VNPs also experience HIV-1-induced downregulation of CD127 (IL-7Rα) in both CD4+ and CD8+ T cell compartments. The downregulation of CD127 was particularly pronounced in the CD8+ T cell compartment, which is in agreement with a previous study showing an expansion of CD8+CD127- effector cells in HIV-1 infection (74). HIV-1 Tat-mediated downregulation of CD127 has also been postulated to result in impaired CD8+ T cell activation and proliferation without apoptosis (75). Nevertheless, in our study, we show for the first time that VNPs demonstrated a positive correlation of CD4 RTE counts with plasma IL-7 level, suggesting a retention of the established pathway (76) of IL-7-dependent homeostatic maintenance of naive T cells. It is noteworthy that decreased plasma IL-7 levels in VNPs are probably not severe enough to hamper the IL-7-driven production of naive CD4+ T cells, where IL-7R downregulation is also relatively moderate. In this regard, the current study suggests that the preserved naive CD4+ T cell compartment in VNPs could be contributed to by an increased thymic output in response to an increased demand for CD4+ T cells (viral targets), driven by T cell activation and a high viral replication. This mechanism however does not preclude a thymic repopulation-independent pathway that may be operational in VNPs to maintain the CD4+ T cell homeostasis.

Another possible explanation of VNP phenotype could be the unique viral signatures associated with the infecting virus. Although viral attenuation seems unlikely due to ongoing high viral replication in VNPs, viral variants with reduced cytopathicity might occur in these individuals. However, genotyping of *vpr* gene, where possible, from a subset of individuals across all groups in our study revealed no group-specific association of mutations linked to reduced cytopathicity or suppressed apoptosis. Further, previous reports have demonstrated that Nef-mediated suppression of immune activation and apoptosis is not enhanced in the VNP phenotype (77). We also studied co-receptor tropism in VNPs to explore the association of tropism shift and thereby potentially distinct pools of infected cells with a high viral replication. We observed no tropism shift across all the study groups. Nevertheless, these data are limited by the lack of *in vitro* studies of viral isolates from VNPs to investigate their cytopathic potential and its role in the lack of CD4+ T cell depletion. Interestingly, a very recent report has demonstrated that *env* clones from VNPs are fully functional and cytopathic (78).

In summation, our study provides a comprehensive assessment of complex mechanisms governing a lack of disease progression in VNPs despite a high viral replication. Intact thymic function, enabling sustained production of naive CD4+T cells and stable central memory CD4+ T cell preservation in VNPs contributes to a maintained CD4+ T cell homeostasis. This allows VNPs to tolerate consequences of continuous viral replication. The study also highlights the role of functional Treg activity present in VNPs due to the preservation of CD4+ T cell counts and associated with activation of CD4+ T cells. The disparate regulation of CD8+ effector T cell generation, manifested as diminution of terminally differentiated effector subset exclusively in VNPs together with stable CD8+ T_CM_ counts, presents a rationale for future functional studies of the role of CD8+ effector T cell compartment in viral pathogenesis. Furthermore, although profound downregulation of CD127 (IL-7Rα) and exhaustion of CD8+ effector T cells possibly impair CD8+ T cell cytotoxic function, its impact on observed disparate regulation of CD8+ effector T cell compartment within VNPs remains to be elucidated. The delineation of these signatures in our report presents a strong case for future longitudinal studies that would further explore specific mechanisms of non-progression in these individuals that result in preserved CD4+ T cell count as well as for immune restoration under failing ART.

## Data Availability Statement

All the sequence data generated in the present study were deposited at NCBI GenBank database (https://www.ncbi.nlm.nih.gov/genbank/) under accession #MN275956-MN276008.

## Ethics Statement

The studies involving human participants were reviewed and approved by ICMR-NIRRH Institutional Ethics Committee for Clinical Studies (Project no.:225/2012). The patients/participants provided their written informed consent to participate in this study.

## Author Contributions

VPat and AS were involved in the study design, performing and overseeing laboratory execution, data analysis, and writing of the original manuscript. AS, SS, VPad, and SV enrolled participants and performed the experiments. AS, VPat, and JS designed the genetic characterization study and was executed and analyzed by JS and SB. JS also assisted in the writing of the manuscript. SM was involved in performing Sanger sequencing and analysis. VN and PP assisted with participants' recruitment and clinical history collection. VPat and AS reviewed and edited the manuscript. VPat and AS approved the final manuscript.

### Conflict of Interest

The authors declare that the research was conducted in the absence of any commercial or financial relationships that could be construed as a potential conflict of interest.

## Abbreviations

VNPs: viremic non-progressors
PuPs: putative progressors
SPs: standard progressors
LTNPs: long-term non-progressors
RTEs: recent thymic emigrants
PLHIV: people living with HIV
NHPs: non-human primates.
