# Supplementary material for: Delineation of Homeostatic Immune Signatures Defining Viremic Non-progression in HIV-1 Infection
Source: Front Immunol. 2020 Mar 5;11:182. doi: 10.3389/fimmu.2020.00182 (PMC7066316; doi:10.3389/fimmu.2020.00182)
Supplement: Supplementary file 1 [file Data_Sheet_1.pdf]

Supplementary Material

SUPPLEMENTARY DATA

Methods in genotyping of *vpr* and *env* gene

Preparation of proviral DNA

Genomic DNA was extracted from PBMCs with QIAamp blood DNA mini kit (Qiagen, Valencia, CA) as per manufacturer's instruction. Isolated DNA was assessed for purity and further quantitated spectrophotometrically.

PCR Amplification of *vpr* and *env*:

Extracted genomic DNA was used as the template for amplification of *vpr* and *env* regions using nested PCR protocols. The reaction volume for the 1st round PCR of *vpr* (4066bp) was 50µL wherein ~1µg of template was amplified using 0.4µM each of Pro3F and PolVpuR2 primers, 1X PCR buffer with 2.5mM of $MgCl_2$, 1.6mM of dNTPs Mix, 2.5U of LA Taq DNA polymerase (TaKaRa) and nuclease free H_2O. Cycling conditions used for 1st round PCR were 94^0C for 2 minutes followed by 10 cycles of (94^0C 10 s, 60^0C 30 s, 68^0C 4 min), then 20 cycles of (94^0C 10 s, 55^0C 30 s, 68^0C 4 min) followed by extension at 68^0C for 10 minutes. Nested round PCR was carried out with 5µL of the 1st round product as template for amplification of the *vpr* region (671bp amplicon). The reaction was carried out in a volume of 50µL with 1X PCR buffer, 3.6mM of $MgCl_2$, 1mM of dNTPs mix, 0.4 µM each of VifB and Acc8R primers, 5U of Taq DNA polymerase (Bangalore Genei, India) and nuclease free H_2O. Cycling conditions used were 3 cycles of 94^0C for 1 minute, 55^0C for 1 minute and 72^0C for 1 min 40 seconds, 32 cycles of (94^0C 15s, 55^0C 45s, 72^0C 1 min), followed by extension at 72^0C for 5 minutes. For amplification of *env* region, two alternate approaches were used wherein the first round PCRs were carried out with primers ED3 and ED14 (2005bp) or ED5 and ED12 (1267bp) followed by second round PCRs with primers ED5 and ED33 (825bp amplicon) or ED31 and ED33 (565bp amplicon) respectively. Preparations of PCR master mix as well as cycling conditions were identical to *vpr* second round PCR described above. Detailed description of primers has been provided in supplementary Table 3. Final PCR products were run on 2% Agarose gel and examined under UV gel documentation system. PCR products were further purified with Nucleospin PCR gel purification kit (Machery Nagel) as per manufacturer's instructions.

SUPPLEMENTARY TABLES AND FIGURES

Tables

Supplementary Table 1: HIV-1 infected participants Characteristics

Participants ID	Gender	Age (Years)	Duration of infection (Years)	Absolute CD4+ T cell count	Absolute CD8+ T cell count	Viral load (Log_{10}VL)
VNP1[#]	Female	40	8	629	984	4.7
VNP2	Female	54	7	582	2781	4.6
VNP3	Male	18	18	505	2106	4.5
VNP4[*]	Female	31	14	704	961	4.0
**VNP5**[*#]	Male	49	11	610	1972	5.3
VNP6	Female	35	7	520	946	4.7
VNP7[*]	Female	30	8	754	1454	5.1
**VNP8**[*]	Female	40	8	517	805	4.6
**VNP9**[*#]	Male	32	9	589	1829	4.8
**VNP10**[*$]	Female	35	16	501	2719	5.3
VNP11	Male	43	13	656	1616	4.1
VNP12	Female	43	8	570	2675	4.2
VNP13	Female	46	10	899	622	4.8
VNP14	Female	45	12	805	1282	4.5
VNP15	Female	44	10	618	1057	4.4
VNP16[*]	Female	40	12	645	1328	4.6
**VNP17**[*]	Female	37	12	866	1818	4.4
VNP18[#]	Female	40	9	910	962	4.2
**PuP19**[*#]	Male	21	2	520	1020	4.5
**PuP20**[*#]	Male	32	0.8	553	1421	6.0
PuP21	Female	35	1	545	613	4.5
PuP22[#]	Female	27	1	719	979	5.6
PuP23	Female	32	0.6	588	611	3.4
PuP24[*#]	Female	51	2	510	2249	3.6
**PuP25**[*#]	Female	53	1	518	1372	5.0
PuP26[*#]	Male	36	1	710	1520	3.8
PuP27[*#]	Male	59	0.8	641	1334	4.1
**PuP28**[*#]	Male	28	1	630	2001	4.7
PuP29[*]	Female	25	3	514	1850	4.0
PuP30[#]	Male	57	1.5	534	1226	5.5
PuP31[*#]	Male	37	0.5	614	836	4.9
PuP32[#]	Female	45	1	908	585	3.6
VC33[*#]	Female	52	9	540	942	2.8

VC34	Female	43	11	757	1120	1.7
VC35*#	Female	42	14	1469	853	3.0
VC36*#	Male	32	10	505	2260	2.9
VC37*	Female	29	10	501	1717	3.0
VC38#	Male	35	8	900	2115	3.0
VC39	Female	60	8	1253	3071	2.9
VC40	Male	45	24	928	1066	2.2
**SP41*#**	Male	32	2	424	1764	4.6
**SP42*#**	Male	45	0.5	87	943	5.4
**SP43*#**	Male	43	0.6	82	1178	5.6
**SP44*#$**	Male	45	0.5	268	793	4.7
SP45#	Male	52	3	402	860	4.5
SP46*	Male	55	0.5	376	495	4.0
SP47#	Male	30	2	338	1543	4.2
**SP48***	Male	40	1	372	555	4.2

*Represents participants enrolled for *vpr* genotyping

# Represents participants enrolled for *env* genotyping

Bold represents participants detected with known mutation

$Represents participants with more than one mutation

Supplementary Table 2: Summary of genotyping data in the study

	VNP	PuP	SP	VC
***vpr* gene mutations**				
Q3R (Amino acid substitution at position 3)	1/8	1/9	0/6	0/4
R77Q (Amino acid substitution at position 77)	5/8	4/9	5/6	0/4
F72L (Amino acid substitution at position 72)	1/8	0/9	0/6	0/4
Mutation in SRIG motif	1/8	0/9	0/6	1/4
W54G, R36W, I64E, L67A, I70S, L64PAR and Q65R	0/8	0/9	0/6	0/4
**No tropism shift (CCR5 (R5) tropism to CXCR4 (X4) tropism) was observed in any infected individual**				
**No distinct N-linked glycosylation pattern was observed in any infected group**				

Supplementary Table 3: Primer sequences for genotyping of *vpr* and *env* genes

Primer Name	Sequence (5'-3')	HXB2 coordinates
Pro3F	AGANCAGAGCCAACAGCCCCACCA	2143-2166
PolVpuR2	CTCTCATTGCCACTGTCTTCTGCTC	6201-6231
VifB	ATATAGCACACAAGTAGACCCT	5319-5340
Acc8R	TCTCCGCTTCTTCCTGCCATAG	5968-5989
ED3	TTAGGCATCTCCTATGGCAGGAAGAAGCGG	5956–5985
ED14	TCTTGCCTGGAGCTGTTTGATGCCCCAGAC	7960–7931
ED5	ATGGGATCAAAGCCTAAAGCCATGTG	6556–6581
ED12	AGTGCTTCCTGCTGCTCCCAAGAACCCAAG	7822–7792
ED31	CCTCAGCCATTACACAGGCCTGTCCAAAG	6816–6844
ED33	TTACAGTAGAAAAATTCCCCTC	7359–7380

Figures

(A)

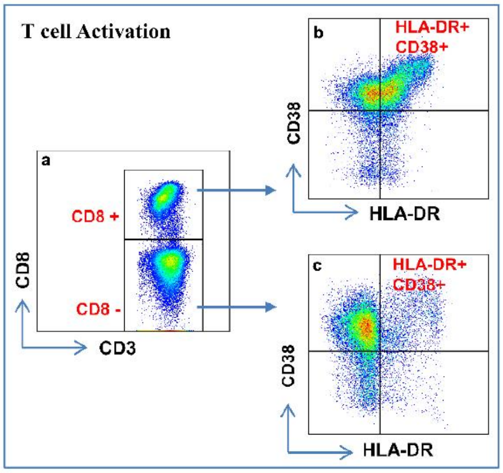

(B)

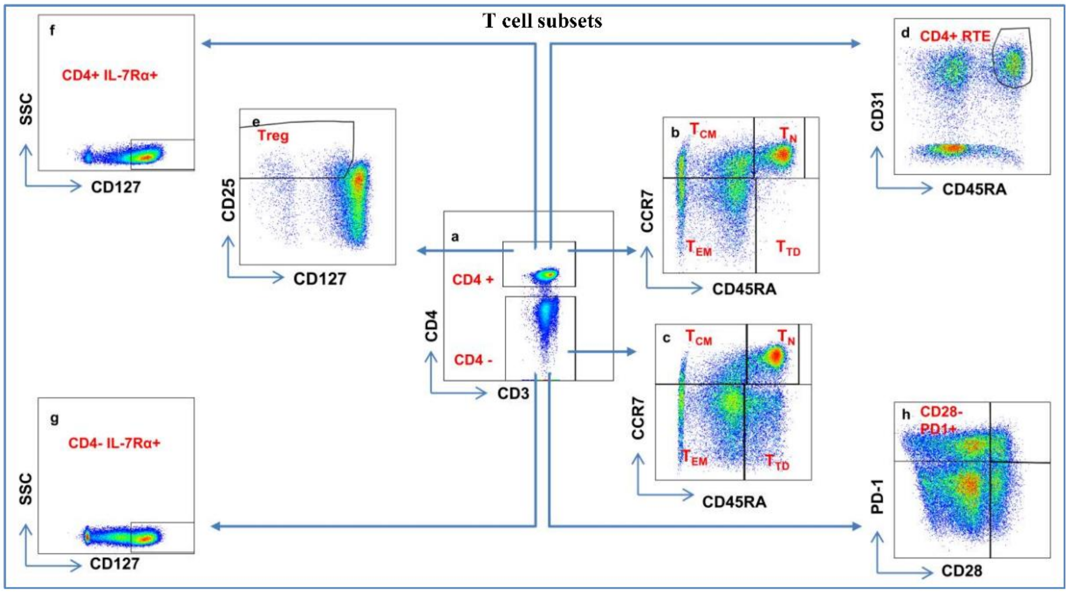

Supplementary Figure S1: Representative flow cytometric gating strategy for CD4+ and CD8+ T cell subsets. (A) First, **(a)** CD8+ and CD4+ (CD8-) T cell were identified on the basis of expression of CD3 and CD8 markers. Next, activated CD8+ **(b)** and CD4+ **(c)** T cells were gated based on co-expression of HLA-DR and CD38 in upper left quadrant. **(B)** CD4+ and CD8+ (CD4-) T cell were first identified on the basis of expression of CD3 and CD4 markers **(a)**. Based on surface expression of CD45RA and CCR7, CD4+ T cell compartment **(b)** and CD8+ T cell compartment **(c)** was distinguished into naïve (T_N), central memory (T_{CM}), effector memory (T_{EM}) and Terminally differentiated (T_{TD}) T cell subsets. Surface expression of CD45RA and CD31 on CD4+ T cells was used to identify CD4+ recent thymic emigrants (CD4+ RTE) **(d)**. Similarly based on expression of CD25 (IL-2Rα) and CD127 (IL-7Rα) on CD4+ T cell surface Tregs were identified **(e)**. IL-7R expressing CD4+ T cells **(f)** and CD8+ T cells **(g)** were identified based on surface expression of CD127. Also, CD28 and PD-1 expression was used to identify exhaustion of total CD8+ effector population (CD8+CD28-PD1+) **(h)**.

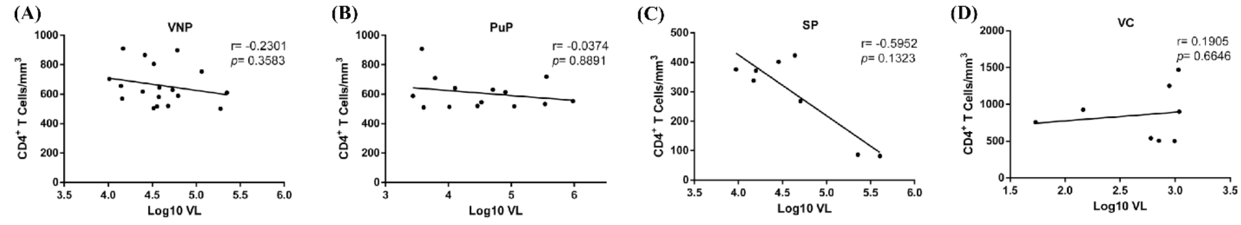

Supplementary Figure S2: CD4+ T cell count and viral load. **(A-D)** Correlation between absolute CD4+ T cell count and plasma viral load (Log_{10}VL) in **(A)**VNPs, **(B)** PuPs, **(C)** SPs and **(D)** VCs. *p* and r values for associations were determined by Spearman's correlation test, with linear regression shown as line. Significant *p* values (*p*<0.05) are in bold.

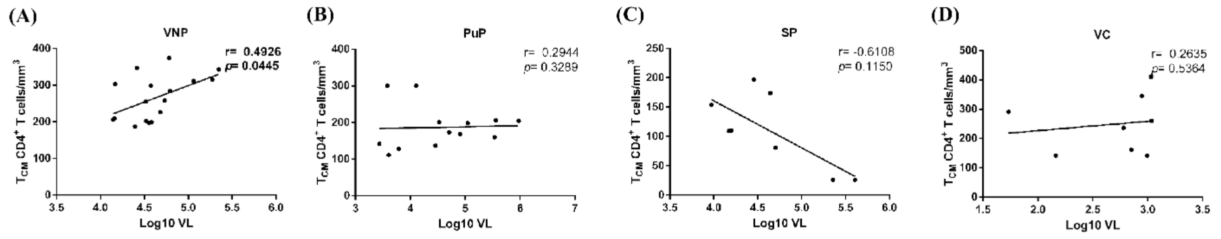

Supplementary Figure S3: Correlation between CD4+ T_{CM} count and plasma viral load in **(A)** VNPs, **(B)** PuPs, **(C)** SPs and **(D)** VCs. *p* and r values for associations were determined by Spearman's correlation test, with linear regression shown as line. Significant *p* values (*p*<0.05) are in bold.

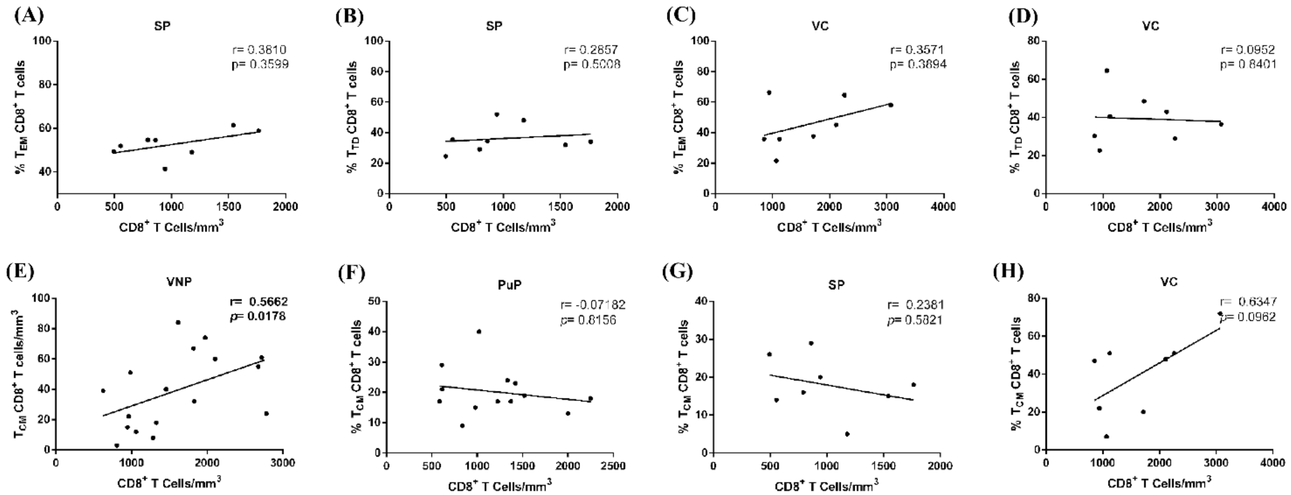

Supplementary Figure S4: Correlation of different CD8+ T cell subset with absolute CD8+ T cell counts. (A-D) Correlation of CD8+ T_{EM} and CD8+ T_{TD} cells frequency with absolute CD8+ T cell counts respectively in **(A, B)** SPs and **(C, D)** VCs. **(E-H)** Correlation of CD8+ T_{CM} cells count with absolute CD8+ T cell counts in **(E)** VNPs, **(F)** PuPs, **(G)** SPs and **(H)** VCs. *p* and r values for associations were determined by Spearman's correlation test, with linear regression shown as line. Significant *p* values ($p<0.05$) are in bold.

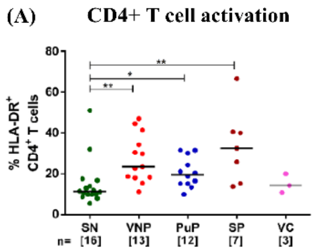

(B) Association between Viral load and CD4+ T cell activation

	CD4+ T cell activation (HLADR+)
**VNP**	**r = 0.5786; *p* = 0.0238**
**PuP**	r = 0.4196; *p* = 0.1745
**SP**	r = -0.0714; *p* = 0.8790
**VC#**	-

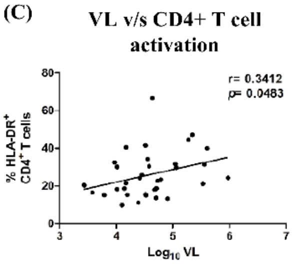

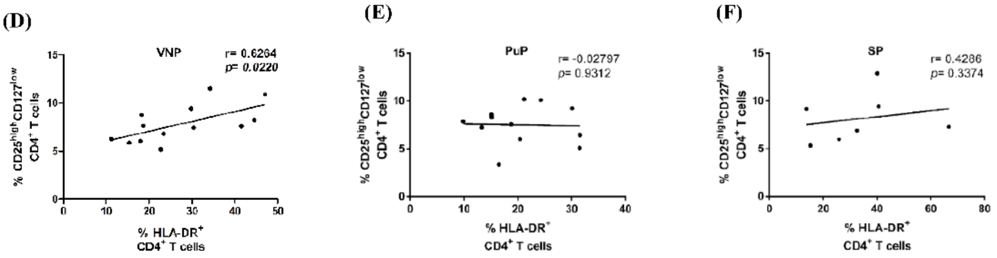

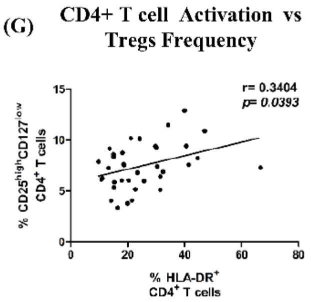

(H) Association between CD4+ T_{CM} frequency and CD4+ T cell activation

	CD4+ T cell activation (HLADR+)
**VNP**	**r= 0.7203; *p*= 0.0082**
**PuP**	r= 0.04545; *p*= 0.8944
**SP**	r= 0.4643; *p*= 0.2939
**VC#**	-

Supplementary Figure S5: CD4+ T cell activation (CD4+ HLADR+) data based on only HLADR expression. (A) Frequency of activated CD4+ T cells. **(B)** Correlation of CD4+ cell activation with plasma viral load in VNPs, PuPs, and SPs **(C)** Cumulative Correlation analysis across all HIV-1 infected individuals of CD4+T cell activation with plasma viral load. **(D-F)** Association of CD4+ T cell activation with Treg frequency respectively in **(D)** VNPs, **(E)** PuPs and **(F)** SPs. **(G)** Cumulative Correlation analysis across all HIV-1 infected individuals of CD4+T cell activation with Treg frequency. **(H)** Correlation of CD4+ cell activation with CD4+ central memory frequency in VNPs, PuPs, and SPs. Comparison between groups were calculated by Mann-Whitney non-parametric test (*p <0.05; **p < 0.01; ***p < 0.001). p and r values for associations were determined by Spearman's correlation test, with linear regression shown as line. Significant (p<0.05) values are in bold. #T cell activation data were available for only 3 VCs and were not further analysed (B and H).
